# Anticancer Efficacy of Targeted Shikonin Liposomes Modified with RGD in Breast Cancer Cells

**DOI:** 10.3390/molecules23020268

**Published:** 2018-01-29

**Authors:** Xianchun Wen, Jiping Li, Defu Cai, Liling Yue, Qi Wang, Li Zhou, Li Fan, Jianwen Sun, Yonghui Wu

**Affiliations:** 1 Public Health College, Harbin Medical University, Harbin 150081, China; wen@qmu.edu.cn (X.W.); lijipinglyh@163.com (J.L.); sjw0451@foxmail.com (J.S.); 2 Research Institute of Medicine & Pharmacy, Qiqihar Medical University, Qiqihar 161006, China; cai@qmu.edu.cn (D.C.); yuell1025@126.com (L.Y.); mianyiwq@126.com (Q.W.); zhou641122@sohu.com (L.Z.); fan123456li@126.com (L.F.)

**Keywords:** shikonin, integrin α_v_β_3_, liposomes, apoptosis, migration, targeted therapy

## Abstract

Shikonin (SHK) has been proven to have a good anti-tumor effect. However, poor water solubility and low bioavailability limit its wide application in clinical practice. In this study, to overcome these drawbacks, RGD-modified shikonin-loaded liposomes (RGD-SSLs-SHK) were successfully prepared. It exhibited excellent physicochemical characteristics including particle size, zeta potential, encapsulation efficiency, and delayed release time. Meanwhile, the targeting activity of the RGD-modified liposomes was demonstrated by flow cytometry and confocal microscopy in the α_v_β_3_-positive MDA-MB-231 cells. Besides exhibiting greater cytotoxicity in vitro, compared with non-targeted shikonin-loaded liposomes (SSLs-SHK), RGD-SSLs-SHK could also evidently induce apoptosis by decreasing the expression of Bcl-2 and increasing the expression of Bax. It could also inhibit cell proliferation, migration, invasion, and adhesion by reducing the expression of MMP-9 and the level of NF-κB p65, but did not affect the expression of MMP-2 in the MDA-MB-231 cells. Therefore, these findings indicated that the strategy to use RGD-modified liposomes as carriers for targeted delivery of shikonin is a very promising approach to achieve breast cancer targeted therapy.

## 1. Introduction

Breast cancer is the most prevalent malignant tumor among women worldwide and second only to lung and bronchus cancer as the major cause of cancer-related death [1,2]. Based on global cancer epidemic statistics estimates, about 1.7 million new breast cancer cases were diagnosed and 521,900 died in 2012 worldwide [3]. This represents about 12% of all new cancer cases (14.1 million). Breast cancer alone accounts for 25% of all cancer cases and 15% of all cancer deaths among females. More developed countries account for about one-half of all breast cancer cases and 38% of deaths [3]. Currently, surgery followed by chemotherapy is the main routine method to inhibit tumor cell growth, proliferation, migration and adhesion, and induce its apoptosis [4,5]. However, the drugs employed in chemotherapy are similarly characterized by poor solubility, significant toxicity, non-targeting, short residence time, and other unpredictable side effects. Among several mechanisms of medication, several studies have demonstrated that shikonin may be a candidate of therapeutic agent in cancer treatment [6].

**Figure 1:**
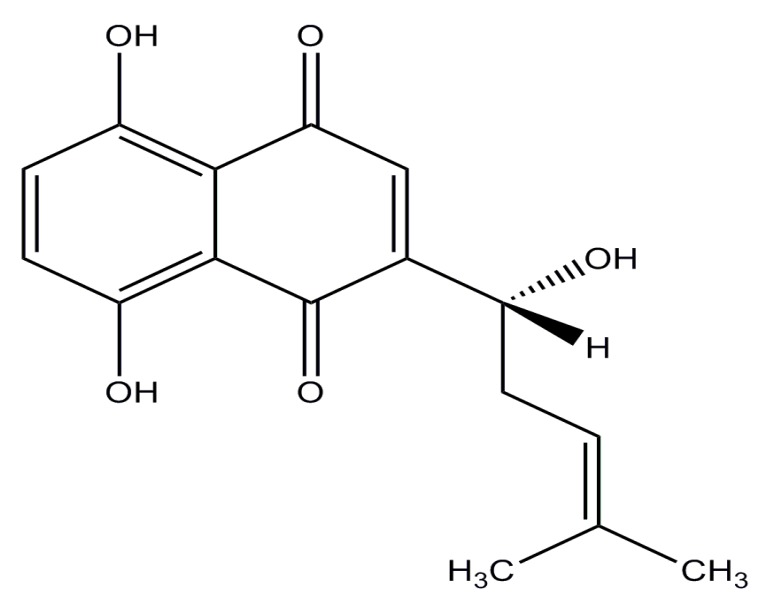
The structure of shikonin (C_16_H_16_O_5_; molecular weight: 288.30).

Shikonin (SHK), a highly lipophilic naphthoquinone isolated from *Lithospermum erythrorhizon*, which has long been used as a traditional Chinese medicine for the treatment of carbuncles, measles, macular eruptions, and sore throat [7,8] (its structure is shown in Figure 1). Previous studies have shown that SHK possesses significant anti-tumor effect on many cancers, including breast cancer, melanoma, gastric cancer, hepatocellular cancer, glioblastoma, and thyroid carcinoma. The anticancer mechanisms of SHK have been widely studied, which are mainly reflected in the following aspects: (1) SHK could inhibit proliferation by reducing tumor-derived exosomes and depressing estrogen signaling [9,10]; (2) it could suppress migration and invasion by inhibiting epithelial mesenchymal transition (EMT) and matrix metalloproteinase-9 (MMP-9) [11,12]; (3) it could induce apoptosis and necroptosis by mediating apoptosis-related proteins expression and reducing reactive oxygen species (ROS) [13,14]; and (4) it could influence transcription by regulating signal transducer and activator of transcription3 (STAT3), focal adhesion kinase (FAK), and steroid receptor coactivator (Src) activation [15]. The multiple inhibitory effects of SHK on tumors suggest that it may be an excellent candidate for cancer therapy. However, SHK has not yet been applied to cure cancer in clinical settings due to its poor aqueous solubility, high toxicity, and extensive first-pass elimination, features which are similar to those of many other chemotherapeutic drugs [16]. Thus, to overcome these drawbacks, a novel targeted drug delivery system for SHK-based therapy is extremely desirable.

Nowadays, many nano-drug delivery systems such as solid lipid nanoparticles, micelles, liposomes, microemulsions, and dendrimers have already been introduced into the clinical application [17]. Among these, the liposomes have been extensively studied as a platform for cancer chemotherapy by virtue of their high biocompatibility and biodegradability [18]. Drug-loaded liposomes (in the size range of 20–200 nm) can be passively accumulated in tumor tissue via the “enhanced permeability and retention (EPR)” effect [19]. However, these liposomes cannot achieve the best therapeutic effect as expected, as they may be rapidly eliminated by the reticuloendothelial system (RES). Thus, constant efforts have been devoted to exploit active targeted systems, which can transport drug to the tumor tissue by an interaction between the conjugated ligand of the liposomes surface and the receptor on cancer cells surface [20]. Among them, integrin α_v_β_3_ receptor which could be specifically and selectively recognized by peptide motif RGD is overexpressed in some tumor cells such as breast cancer, lung cancer, and activated vascular endothelial cells, while it is rarely expressed in other endothelial cells and most noncancerous organs [21]. Meanwhile, some research has shown that α_v_β_3_ receptor could be overexpressed on the surface of MDA-MB-231, MDA-MB-435, U87MG, and HepG2 cells, while it is not expressed or low expressed on that of MCF-7, LO2, and M21L cells [18,22,23]. RGD, which is a cyclic peptide containing Arg-Gly-Asp, bears strong affinity for integrin α_v_β_3_ receptor on the tumor cells surface, and liposomes modified with RGD have been proven to have obvious effect as anticancer drug delivery systems relative to non-targeted liposomes [24]. Some studies have showed that the use of active targeted RGD ligands could cause many liposomes to accumulate in the tumors [25,26]. However, the application of RGD-modified liposomes as carriers for targeted delivery of SHK against tumor has not been reported yet.

The purpose of this study was to first construct the SHK-loaded liposomes modified with RGD (RGD-SSLs-SHK), and to evaluate their physicochemical characteristics including the particle size, zeta potential, morphology, encapsulated efficiency and drug release. The in vitro cellular uptake of coumarin-6 loaded RGD modification liposomes in the MDA-MB-231 and MCF-7 cells was investigated via flow cytometry and confocal microscopy analysis. Meanwhile, the anticancer effects of RGD-SSLs-SHK were verified by cytotoxicity, migration, invasion, adhesion, and apoptosis in vitro. Finally, to explore therapeutic efficiency and the mechanism of RGD-SSLs-SHK in depth, the expression of carcinogenic-related proteins (MMP-2, MMP-9, NF-κB p65, Bcl-2, and Bax) were assayed by Western blotting in the MDA-MB-231 cells. Altogether, the results obtained would support the hypothesis that RGD-SSLs-SHK could play an important role in targeting integrin α_v_β_3_-overexpressed tumors and enhancing the anti-tumor efficacy.

## 2. Results and Discussion

### 2.1. Preparation and Characterization of Liposomes

**Figure 2:**
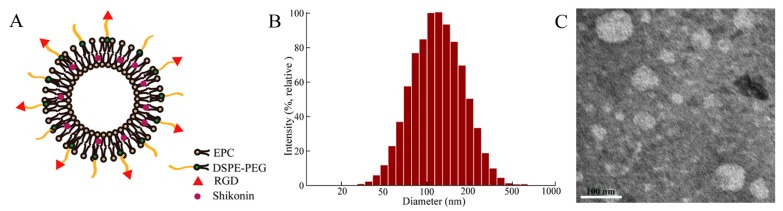
Schematic illustration of RGD-modified shikonin-loaded liposomes (RGD-SSLs-SHK) (**A**); The particle size distribution of RGD-SSLs-SHK (**B**); and TEM image of RGD-SSLs-SHK (**C**).

All liposomes were prepared by the thin-film hydration method that had been proverbially used (Figure 2A). The average particle sizes of these liposomes were less than 125 nm with a polydispersity index (PDI) about 0.21 (Figure 2B and Table 1), which played an important role in the distribution of drugs in vivo. It has been reported that the liposomes ranging 100–200 nm in diameter were significantly accumulated in the tumor tissue in view of EPR effect [27,28]. The zeta potentials of SSLs-SHK and RGD-SSLs-SHK were -16.32 ± 1.45 and -15.37 ± 0.91 mV (Table 1), respectively. The TEM image revealed that the morphologies of most liposomes were in near spheroid-like vesicles near 100 nm (Figure 2C), which were consistent with the value obtained by the dynamic light scattering (DLS). The samples were both unilamellar without aggregation. Encapsulation efficiencies (EE) of all liposomes were higher than 92% (Table 1). These results demonstrated that the RGD peptide modification did not influence physicochemical properties of liposomes.

### 2.2. In Vitro Release Behavior of Liposomes

**Figure 3:**
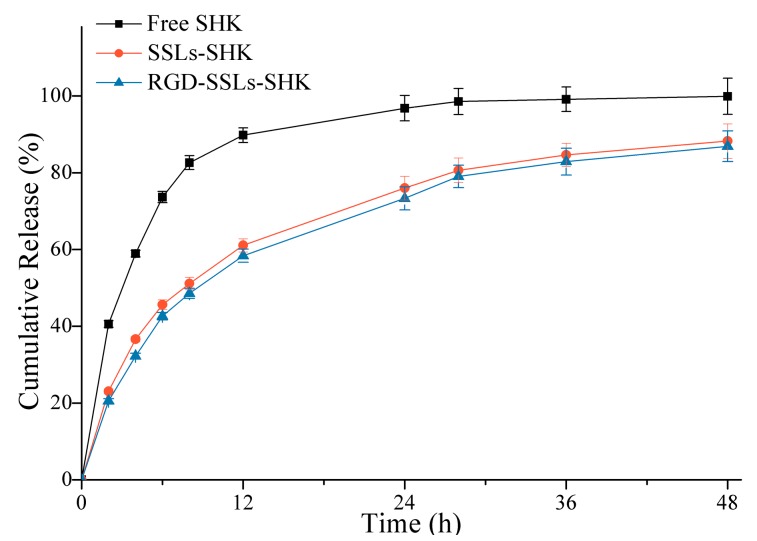
In vitro cumulative release of SSLs-SHK, RGD-SSLs-SHK, and free SHK at pH 7.4 for 48 h.

To verify release behavior of liposomes, release experiments of SSLs-SHK, RGD-SSLs-SHK, and free SHK were investigated in PBS at pH 7.4 for 48 h using a dialysis method (Figure 3). Compared to the fast release of free SHK with 82.62% ± 2.76% in the initial 8 h, SSLs-SHK and RGD-SSLs-SHK exhibited similar controlled gradual release characteristics. SHK from SSLs-SHK and RGD-SSLs-SHK was only 51.14% ± 1.98% and 47.56% ± 2.36% released in the same period, respectively. At 24 h, the release rate of free SHK was approximately 98%, while SHK release from both liposomes was only approximately 75%. The results suggested that SHK was released slowly from the liposomes. In addition, release behavior of SHK from the both liposomes had no significant difference under the same condition, which was beneficial for the following investigation of the two liposomal systems in cells. This result also indicated that the superior lipid membrane could play an important role in avoiding the escape of encapsulated drug as a natural barrier. Simultaneously, the release behavior of liposomes was favorable for reducing drug leakage in circulation before delivering drug to target site.

### 2.3. In Vitro Cellular Uptake

**Figure 4:**
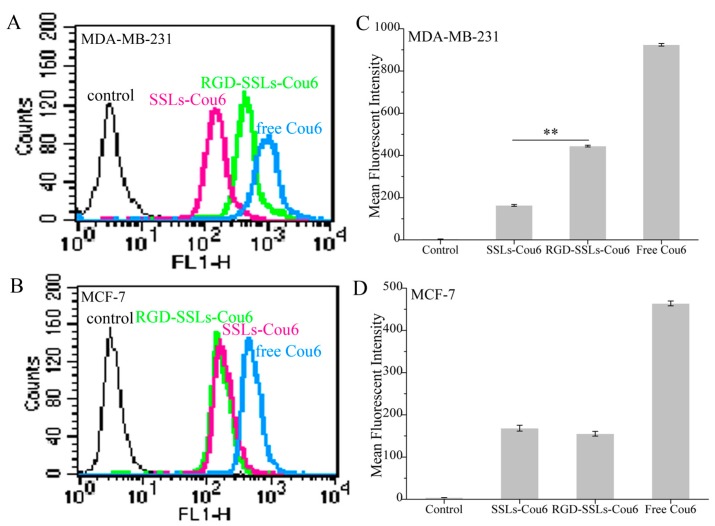
Flow cytometry profiles of: MDA-MB-231 (**A**); and MCF-7 cells (**B**) after treatment with SSLs-Cou6, RGD-SSLs-Cou6, and free Cou6 containing medium at concentration of 50 ng/mL for 1 h. Mean fluorescence intensity of: MDA-MB-231 (**C**); and MCF-7 cells (**D**) were assayed by flow cytometry experiments. ** *p* < 0.01.

To evaluate the capacity of RGD-modified liposomes to boost intracellular delivery, the cellular uptakes of different coumarin-6 (Cou6) formulations in the α_v_β_3_-positive MDA-MB-231 and α_v_β_3_-negative MCF-7 cells were assayed using flow cytometry and laser confocal microscopy. Flow cytometry analyses were performed on the MDA-MB-231 cells and MCF-7 cells after incubation for 1 h with SSLs-Cou6, RGD-SSLs-Cou6, and free Cou6. The cellular uptake of RGD-SSLs-Cou6 in the MDA-MB-231 cells embodied higher fluorescence intensity than that of SSLs-Cou6 (*p* < 0.01) (Figure 4A,B), whereas no significant difference was observed in the MCF-7 cells for the both liposomes (Figure 4C,D). Meanwhile, a positive control of free Cou6 showed the highest cellular Cou6 level, as it could directly cross over the cell membranes into the cells with transient release process, leading to the greatest extent of cellular accumulation [29].

**Figure 5:**
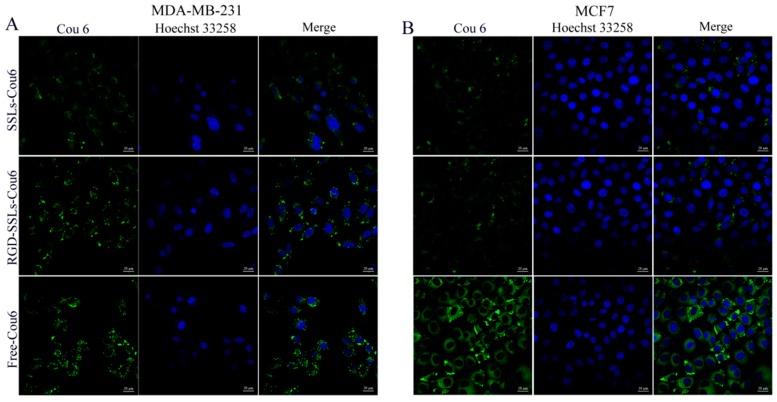
Determination of the cellular uptake via laser confocal microscopy. SSLs-Cou6, RGD-SSLs-Cou6, and free Cou6 were incubated with: MDA-MB-231 cells (**A**); and MCF-7 cells (**B**) at concentration of 50 ng/mL for 1 h. The cell nucleus was stained with Hoechst33258 (blue).

Laser confocal microscopy images obtained similar results. After the same incubation time, free Cou6 showed the highest fluorescence intensity, as mentioned above. The uptake of RGD-SSLs-Cou6 in the MDA-MB-231 cells exhibited higher fluorescence intensity in cytoplasm than that of SSLs-Cou6 (Figure 5A), while both liposomes in the MCF-7 cells were almost the same (Figure 5B). These results demonstrated that the RGD-modified liposomes could remarkably strengthen the intracellular uptake through receptor-mediated endocytosis, and have high selectivity towards cancer cells [30]. This is mainly attributed to the ligand feature of RGD, which could better recognize and react with α_v_β_3_ receptor, thereby improving its intracellular uptake [21].

### 2.4. In Vitro Cytotoxicity Investigation

**Figure 6:**
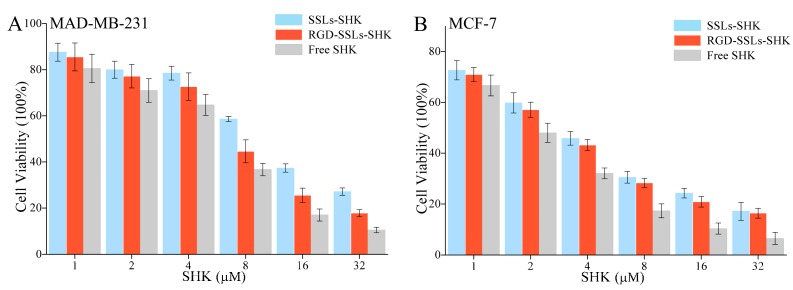
In vitro cytotoxicity effects of different Shikonin (SHK) formulations on: MDA-MB-231 cells (**A**); and MCF-7 cells by MTT assay (**B**). Both cells were treated with SSLs-SHK, RGD-SSLs-SHK, and free SHK at various concentrations of SHK for 24 h.

To estimate anticancer effects in vitro, the cytotoxicity of different SHK formulations on the MDA-MB-231 and MCF-7 cells with various concentrations of SHK (1, 2, 4, 8, 16, and 32 µM) for 24 h were measured using MTT method. The results indicated that SSLs-SHK, RGD-SSLs-SHK, and free SHK could all inhibit cells proliferation in a concentration-dependent manner (Figure 6A,B). The cytotoxicity of free SHK was the strongest on the MDA-MB-231 and MCF-7 cells (IC_50_: 4.92 ± 0.29 μM and 1.90 ± 0.11 μM) compared to those of SSLs-SHK (IC_50_: 10.92 ± 1.03 μM and 3.34 ± 0.18 μM) and RGD-SSLs-SHK (IC_50_: 7.16 ± 0.62 μM and 2.96 ± 0.12 μM) (Table 2). The above results suggested that free SHK could rapidly pass through cytomembrane directly into intracellular with the drug short release process, while the liposomes were internalized via endocytosis with a certain amount of time, and then possibly underwent a persistent release process [31]. However, RGD-SSLs-SHK displayed higher cytotoxic capacity for the MDA-MB-231 cells than SSLs-SHK (*p* < 0.01), and the inhibition of the both liposomes on the MCF-7 cells had no significant difference (Table 2). These results demonstrated that the uptake of RGD-SSLs-SHK was chiefly mediated by the special binding between RGD and α_v_β_3_ receptors overexpression on the MDA-MB-231 cells. Hence, RGD-modified liposomes could deliver more SHK into α_v_β_3_-overexpressed cancer cells in certain time and augmented its anticancer effect [32]. Meanwhile, the inhibition effects of RGD-SSLs-SHK and SSLs-SHK on the MCF-7 cells proliferation were also very significant, but α_v_β_3_ receptor is not expressed or low expressed in the MCF-7 cells, thus MCF-7 cells were used as a negative control.

### 2.5. In Vitro Apoptosis-Promoting Effect of RGD-SSLs-SHK

**Figure 7:**
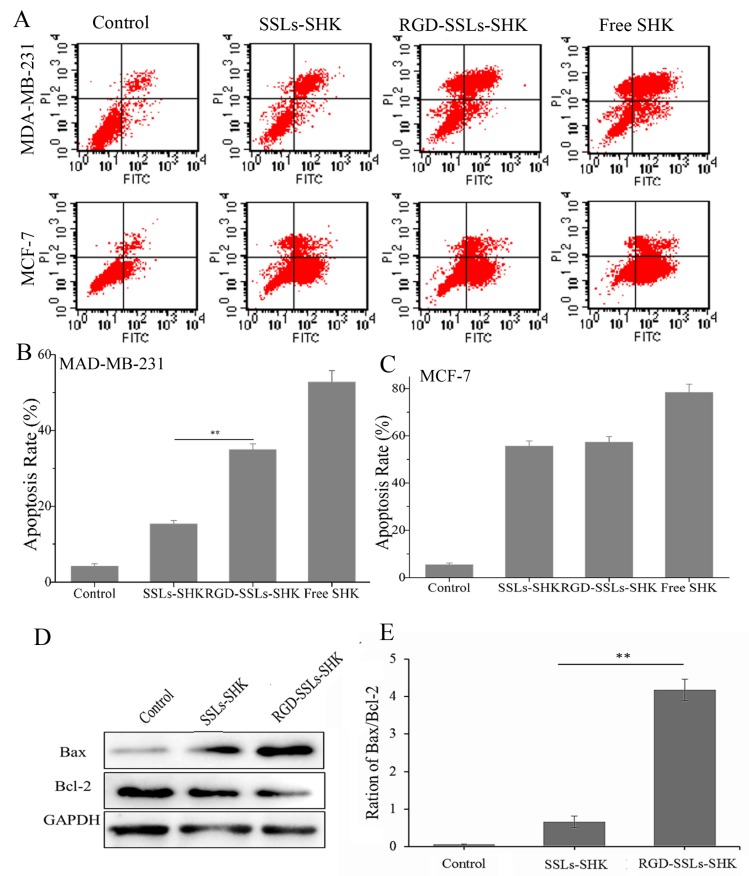
In vitro apoptotic analysis of Control, SSLs-SHK, RGD-SSLs-SHK, and free SHK on the MDA-MB-231 and MCF-7 cells via flow cytometry (**A**); The comparison of apoptosis rate of control, SSLs-SHK, RGD-SSLs-SHK, and free SHK in: MDA-MB-231 cells (**B**); and MCF-7 cells (**C**); Western blot analysis of Bax and Bcl-2 expression after the MDA-MB-231 cells were treated with SSLs-SHK and RGD-SSLs-SHK for 24 h (**D**); The ratio of Bax/Bcl-2 was calculated according to the band intensity in each group (**E**). ** *p* < 0.01.

Furthermore, to evaluate the apoptosis-inducing effect of RGD-modified liposomes against breast cancer cells, the degree of apoptosis was measured by flow cytometry after the cells were treated with different SHK formulations at the same concentration (2 μM) for 24 h and stained with Annexin V-FITC/PI. As shown in Figure 7A,B, the apoptosis rate of the control, SSLs-SHK, RGD-SSLs-SHK, and free SHK group were 4.25% ± 0.64%, 15.38% ± 0.85%, 34.97% ± 1.48%, and 52.8% ± 2.61% in the MDA-MB-231 cells, respectively. As a positive control, free SHK exhibited the maximal apoptosis rate, which was in accordance with cytotoxicity results. The apoptosis rate in RGD targeting group was obviously increased compared with passive SSLs-SHK group in the MDA-MB-231 cells (*p* < 0.01) (Figure 7B). No evident difference was observed in the MCF-7 cells as a negative control for the two type liposomes (Figure 7C). The results indicated that the liposomes modified with RGD could lead to great activities in the induction of tumor cell apoptosis, which were consistent with the enhancement of cellular uptake described above, implying the association between intracellular drug level and tumor suppression efficacy.

To further elucidate the apoptosis-inducing mechanism of RGD-SSLs-SHK, the expression of the Bcl-2 family members Bcl-2 and Bax in the MDA-MB-231 cells were assayed by Western blotting. We observed a notable reduction of Bcl-2 expression after the both liposomes treatment, accompanied by increases of Bax expression, thereby the ratio of Bax/Bcl-2 was evidently increased in the MDA-MB-231 cells (Figure 7D,E). In addition, the effect of RGD-SSLs-SHK-induced apoptosis was significantly stronger than that of SSLs-SHK’s in the MDA-MB-231 cells (*p* < 0.01) (Figure 7E). As we all know, mitochondria play an important role in the signal pathway of apoptosis. The members of the Bcl-2 family, as major cell survival and cell death regulators, were the main way to regulate the signaling pathway of mitochondria apoptosis [33]. Bcl-2 acts as an anti-apoptotic protein, while Bax acts as a pro-apoptotic protein, and the balance of Bcl-2 and Bax is closely associated with apoptosis [34]. Our results showed that RGD-SSLs-SHK could cause an obvious decline of anti-apoptotic Bcl-2 and an increase of pro-apoptotic Bax, suggesting that the regulation of RGD-SSLs-SHK on Bcl-2 and Bax expression may be a major way of inducing apoptosis in the MDA-MB-231 cells.

### 2.6. In Vitro Inhibition Effects of RGD-SSLs-SHK on Migration, Invasion, and Adhesion 

**Figure 8:**
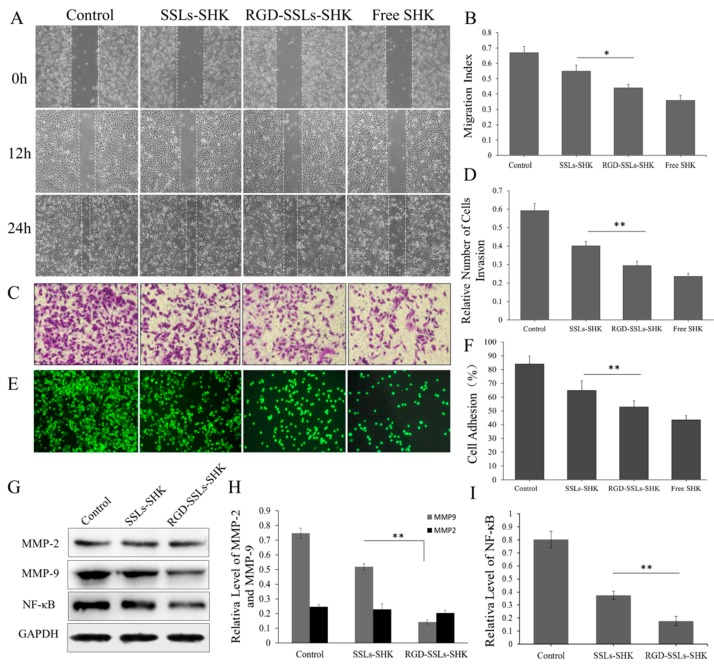
In vitro inhibitory effects of SSLs-SHK, RGD-SSLs-SHK, and free SHK on: migration (**A**); invasion (**C**); and adhesion (**E**) in the MDA-MB-231 by wound healing assay, Transwell invasion, and adhesion assay, respectively. Statistical analysis of: migration index (**B**); cell invasion (**D**); and cell adhesion (**F**) in the MDA-MB-231 cells; Western blot assay results of MMP-2, MMP-9, and NF-κB p65 after the MDA-MB-231 cells was treated with SSLs-SHK and RGD-SSLs-SHK for 24 h (**G**). Analytic results of the band intensity values of: MMP-2 and MMP-9 (**H**); and NF-κB p65 (**I**). * *p* < 0.05, ** *p* < 0.01.

The effects of the liposomes on the migration of the MDA-MB-231 cells were evaluated by scratch wound healing assays. As observed in the images from the wound healing assays, the migration indexes of control, SSLs-SHK, RGD-SSLs-SHK, and free SHK were 0.67 ± 0.04, 0.55 ± 0.03, 0.44 ± 0.02, and 0.36 ± 0.03, respectively (Figure 8A,B). All SHK formulations failed to close the wound after 12 and 24 h, and a positive control of free SHK displayed strongest metastatic inhibition effect (Figure 8A). Meanwhile, the effective anti-metastatic potential of RGD-SSLs-SHK was notably stronger than that of SSLs-SHK (*p* < 0.05) (Figure 8B).

Similarly, the relative invasion number of the MDA-MB-231 cells was also reduced after RGD-SSLs-SHK treatment for 12 h, as evaluated by Transwell assay (Figure 8C,D). Moreover, the effective anti-invasion capacity of RGD-SSLs-SHK was remarkably stronger than that of SSLs-SHK (*p* < 0.01) (Figure 8D), suggesting that RGD modification could further reduce the motility of the MDA-MB-231 cells. These results clearly demonstrated that SHK could availably inhibit the migration and invasion of tumor cells, and RGD-SSLs-SHK had more targeted potency to the α_v_β_3_-overexpressed MDA-MB-231 cells [35].

Subsequently, the cell adhesion effect of different SHK formulations on the MDA-MB-231 cells was also investigated (Figure 8E,F). The result indicated that the adhesion ratio of free SHK was lowest, as a positive control. However, the adhesion ratio of RGD-SSLs-SHK on the MDA-MB-231 cells was very low compared to that of SSLs-SHK (*p* < 0.01) (Figure 8F), which further validated results of migration and invasion experiment, and better proved the targeting and enhancing anticancer ability of RGD-SSLs-SHK.

To explore the anti-migration, anti-invasion, and anti-adhesion mechanisms of RGD-SSLs-SHK, the expression of matrix metalloproteinase-2 (MMP-2), matrix metalloproteinase-9 (MMP-9), and the level of NF-κB p65 in the MDA-MB-231 cells after treatment with SSLs-SHK and RGD-SSLs-SHK (SHK concentration: 2 µM) were determined by Western blotting. The results indicated that both liposomes obviously decreased MMP-9 expression and the level of NF-κB p65, whereas MMP-2 expression was not affected (Figure 8G–I). Meanwhile, the inhibitory effects of RGD-SSLs-SHK on MMP-9 and NF-κB p65 were notably strong compared with that of SSLs-SHK (*p* < 0.01) (Figure 8H,I). MMP-2 and MMP-9, as important members of the MMPs family, are commonly associated with tumor cell migration, invasion, and adhesion in various human cancers, due to their capacities to degrade extracellular matrix (ECM), thereby facilitating cell motility [36]. Moreover, the expression of MMP-2 and MMP-9 also depends on NF-κB, besides other cells signaling pathways. NF-κB is an important transcription factor involved in regulating various transcriptional genes or protein expression including ICAM-1, VCAM1, MMP-2 and MMP-9 [35,37]. It is reported that inhibition of NF-κB activity could decrease the invasion of cancer cells by reducing MMP-9 expression [38]. The above results demonstrated that the RGD-SSLs-SHK may play a role in inhibiting migration, adhesion, and invasion by reducing the levels of MMP-9 and NF-κB p65 in the MDA-MB-231 cells. Furthermore, our results were consistent with previous studies [35,36,37,38], and may also provide scientific evidence for the anticancer mechanism of SHK.

## 3. Materials and Methods

### 3.1. Materials

Shikonin (assay 99.8%) was obtained from Shanghai yuanye Bio-Technology Co., Ltd. (Shanghai, China). DSPE-PEG2000-RGD (purity > 97%) was synthesized by Xi'an ruixi Biological Technology Co., Ltd. (Xi’an, China). DSPE-PEG2000 was purchased from ToYong Biotch Co., Ltd. (Shanghai, China). Egg phosphatidylcholine (EPC, assay 98%) was obtained from LIPOID GmbH (Ludwigshafen, Germany). Cholesterol (Chol), sephadex G-50, coumarin-6 (Cou6), and Hoechst 33258 were all purchased from Sigma-Aldrich (Shanghai, China). Annexin V–FITC/PI apoptosis detection kit was purchased from Beijing ComWin Biotech Co., Ltd. (Beijing, China). 3-(4,5-Dimethylth-iazol-2-yl)-2,5-diphenyltetrazolium bromide (MTT) was obtained from Gibco Brl (Shanghai, China). Rabbit polyclonal anti-human-MMP-2 and rabbit polyclonal anti-human-MMP-9 were obtained from Proteintech Group, Inc. (Chicago, IL, USA). Rabbit polyclonal anti-human-NF-κB p65, mouse polyclonal anti-human-Bcl-2, mouse polyclonal anti-human-Bax, mouse polyclonal anti-GAPDH, rabbit anti-mouse immunoglobulin G (IgG)and goat anti-rabbit IgG were all purchased from Cell Signaling Technology, Inc. (Boston, MA, USA).

### 3.2. Preparation of Liposomes

The liposomes were prepared using the thin-film hydration method [39,40]. Typically, a mixture of EPC, cholesterol, DSPE-PEG2000, and SHK at the molar ratio of 20:10:2:4 was first dissolved in dichloromethane: 100% ethanol (1:3, *v*:*v*) in a round-bottomed flask. Subsequently, the organic solvent was removed using rotary evaporation under vacuum at 37 °C for 1 h, the dried lipid films were hydrated with PBS (pH = 7.4) or ultrapure water at 37 °C till adequate dissolution and then sonicated at 37 °C for 1 h. The suspension was then dispersed and extruded through a polycarbonate membrane of 220 nm followed by gel filtration over a sephadex G-50 column to remove the unencapsulated SHK. Finally, the SHK-loaded liposomes (SSLs-SHK) was formed.

Similarly, A mixture of EPC, cholesterol, DSPE-PEG2000, DSPE-PEG2000-RGD, and SHK in the ratio of 20:10:1.6:0.4:4 formed the RGD-modified liposomes (RGD-SSLs-SHK) by the above procedure. Coumarin 6 (Cou6)-loaded liposomes were prepared instead of SHK by the same protocol, as were the blank liposomes without adding Cou6 or SHK.

### 3.3. Characterization of Liposomes

The average particle sizes and zeta potentials of the various liposomes were determined by the dynamic light scattering (DLS) method using Nicomp 380ZLS Particle Sizing System (PSS, CA, USA). The encapsulation efficiency (EE) was determined by the following formula: EE (%) = 1 − (weight of free SHK/total weight of SHK) × 100%. Briefly, the amount of SHK was determined by high performance liquid chromatography (HPLC) with an UV detector (Waters, Massachusetts, USA), and the mobile phase was isocratic with acetonitrile and double distilled water (75/25, *v*/*v*). At first, 1 of mL sample with acetonitrile was centrifuged at 20,000 rpm for 60 min, the supernatant portion was detected to quantify the total weight of SHK. Another 1 of mL sample diluted with 2% SDS phosphate buffer solution (PBS, pH = 7.4) was centrifuged as the above, the weight of free SHK was detected by HPLC assay. The morphology of liposomes was visualized on transmission electron microscope (Hitachi, Tokyo, Japan) after the sample stained with 2% (*w*/*v*) phosphotungstic acid solution. The above results were repeated three times.

### 3.4. Cell Culture

The human breast cancer cell lines (MDA-MB-231 and MCF-7) were obtained from the Cell Bank of the Chinese Academy of Sciences (Shanghai, China). The MCF-7 cells were grown in high-glucose Dulbecco’s modified Eagle’s medium (DMEM, Hyclone, UT, USA) with 10% fetal bovine serum (FBS, Hyclone, UT, USA) and 1% penicillin–streptomycin solution (Gaithersberg, USA). The MDA-MB-231 cells were cultured in Leibovitz’s L15-medium (L15, Hyclone, UT, USA) containing 10% FBS and 1% penicillin–streptomycin solution. All cell lines were cultured in a humidified incubator at 37 °C. The used cells for the experiment should reach approximately 90% confluency.

### 3.5. In Vitro Release of SHK from Liposomes

Release behavior of SHK was carried out in 2% SDS phosphate buffer solutions at pH 7.4 using a dialysis method in vitro as previously reported [41]. In detail, 1 mL of SSLs-SHK, RGD-SSLs-SHK, and free SHK (SHK concentration: 80 µg/mL) was transferred into a dialysis bag (MWCO = 2000 Da), respectively. Then, the dialysis bag was submerged in 30 mL of release medium at 37 °C for 48 h with horizontal oscillation at 100 rpm. At selected time points, 1 mL of release medium was removed, and then an equal volume of fresh solution was timely added back. The release concentrations of SHK in the release medium were determined by HPLC. Each experiment was performed three times independently.

### 3.6. In Vitro Cellular Uptake 

To investigate the localization of liposomes in vitro, the MDA-MB-231 and MCF-7 cells were seeded into a six-well plate at a density of 4 × 10^5^ cells/well in 2 mL of culture medium. After incubation for 24 h, the cells were treated with SSLs-Cou6, RGD-SSLs-Cou6, and free Cou6 solution at the same final Cou6 concentration of 50 ng/mL diluted with serum-free culture medium. After incubation for 1 h, the collected cells were washed three times with cold PBS, trypsinized, centrifuged, and transferred to a flow tube. The fluorescence intensity of cells was measured by a flow cytometry (Becton Dickinson, NJ, USA) with 10,000 gated events collected, excited at 488 nm and detected at 560 nm.

The absorbability of liposomes in the MDA-MB-231 and MCF-7 cells was also detected via a laser confocal microscopy (Zeiss, Jeantown, Germany). Briefly, the cells were cultured and treated as above method. After incubation for 1 h, the harvested cells were rinsed three times with cold PBS, fixed, and taken photos by a laser confocal microscopy. Each assay was repeated in triplicate.

### 3.7. In Vitro Cytotoxicity of Liposomes Assay

The cytotoxicity of SSLs-SHK, RGD-SSLs-SHK, and free SHK against breast cancer cells were determined by MTT assay. Briefly, the MDA-MB-231 and MCF-7 cells were seeded into 96-well plates at a density of 7 × 10^3^ cells/well and cultured for 24 h at 37 °C, respectively. Then, the medium was removed and the cells were added fresh medium (200 μL) containing SSLs-SHK, RGD-SSLs-SHK, and free SHK at various concentrations of SHK (1, 2, 4, 8, 16, and 32 μM). The cells without treatment were deemed as control. After incubation for 24 h, the cells were incubated with 20 μL of 5 mg/mL MTT solution/well for another 4 h, the medium was removed, and the cells were dissolved with 150 μL DMSO. The absorbance was measured with an enzyme linked immunosorbent assay (ELISA) reader (Tecan, Diken, Austria) at 490 nm. The half-maximal inhibitory concentration (IC_50_) of each treatment was calculated. Each experiment was conducted six times.

### 3.8. In Vitro Apoptosis Measurement

Apoptosis were analyzed via flow cytometry and AnnexinV-FITC/PI staining. The MDA-MB-231 and MCF-7 cells were exposed to SSLs-SHK, RGD-SSLs-SHK, and free SHK (SHK concentration: 2 µM) for 24 h. The harvested cells were washed twice with PBS, and then incubated with Annexin V-FITC and PI for 15 min in the dark. After incubation, the stained cells were measured and analyzed using a flow cytometry.

### 3.9. Cell Migration Assay

Migration experiment was assessed using a monolayer wound healing assay [42]. Briefly, the MDA-MB-231 cells were seeded into 12-well plates at a density of 2.5 × 10^5^ cells/well until they reached approximately 90% confluence. After creating a scratch in the monolayer with a sterile pipette tip, debris was removed by washing the monolayers with PBS, and then the medium was turned into L15 containing 2% FBS with SSLs-SHK, RGD-SSLs-SHK, and free SHK (SHK concentration: 1.5 µM), normal medium as control. After culture for 6, 12, and 24 h, phase contrast microscopy (Olympus, Tokyo, Japan) was used to capture images and image of each scratch area was analyzed by Image J software. Data were quantified through analyzing the areas without covered by cells. The results were taken 0 h as a contrast.

### 3.10. Transwell Invasion Assay

Transwell invasion assay was used to evaluate invasion abilities of the cells. The Transwell membranes were pre-coated with 30 μg/well Matrigel (8.0 Μm pores, Corning, NY, USA). The MDA-MB-231 cells were treated with SSLs-SHK, RGD-SSLs-SHK, and free SHK (SHK concentration: 1.5 µM) for 12 h, and then which were added into the upper chamber at a density of 1 × 10^5^ cells/mL in serum-free medium, and culture medium contained 10% FBS was added to the lower chambers. The non-invading cells were removed from the upper surface of the filter, the invading cells were fixed with 4% paraformaldehyde for 30 min at room temperature and stained with 0.1% crystal violet. After being photographed using phase contrast microscopy, incorporated dye was dissolved in 33% acetic acid. The optical densities of each well were measured by an ELISA reader at 570 nm. Each experiment was performed in triplicate.

### 3.11. Cell–Matrigel Adhesion Assay

The cell adhesion assay was carried out as previous described study [43]. Briefly, the MDA-MB-231 cells (1 × 10^5^ cells/well) were pretreated with SSLs-SHK, RGD-SSLs-SHK, and free SHK (SHK concentration: 1.5 µM) for 24 h. The cells were labeled with 5 µM of Calcein AM for 30 min at 37 °C, and then which were seeded into a 96-well plate precoated with 25 µg/well of Matrigel basement membrane extract. After incubation for another 30 min, the plate was washed 3 times with PBS to remove the unattached cells. After photographed under a fluorescence microscopy (Olympus, Tokyo, Japan), the fluorescence intensity of the attached cells at 520 nm was measured by an ELISA reader. The adhesion rate was calculated on the assumption that 200 µL of Calcein-AM labeled cell suspension represents 100%, and which was calculated according to the formula below: Adhesion rate (%) = the mean fluorescence value of samples/the mean fluorescence value of control group × 100%. Each experiment was repeated at least three times.

### 3.12. In Vivo Western Blotting Analysis

Western blotting technology was used to assay the expression of related protein. MDA-MB-231 cells were pretreated with SSLs-SHK and RGD-SSLs-SHK (SHK concentration: 2 µM) for 24 h. The cells were collected and lysed, and the proteins were resolved by SDS-PAGE and immunoblotted. The immunoblotted were performed with rabbit polyclonal anti-human-MMP-2 (dilution ratio 1:500), rabbit polyclonal anti-human-MMP-9 (dilution ratio 1:500), rabbit polyclonal anti-human-NF-κB p65 (dilution ratio 1:500), mouse polyclonal anti-human-Bcl-2 (dilution ratio 1:800), mouse polyclonal anti-human-Bax (dilution ratio 1:800), and mouse polyclonal anti-GAPDH antibodies (dilution ratio 1:3000) as an internal control, and then were washed three times with TBST before adding the secondary antibodies (rabbit anti-mouse IgG and goat anti-rabbit IgG; dilution ratio 1:3000). The immunoreactivity was detected using enhanced chemiluminescence.

### 3.13. Statistical Analysis

The data were presented as the mean ± SD and analyzed by SPSS 19.0 statistical software. Statistical comparisons were performed by one-way ANOVA and Student’s non-paired *t*-test. Data with *p*-value < 0.05 or 0.01 were considered statistically significant.

## 4. Conclusions

In this study, SHK-loaded liposomes modified with RGD (RGD-SSLs-SHK) were successfully constructed to achieve targeted delivery of SHK into cancer cells. RGD-SSLs-SHK had exhibited great physicochemical properties, which showed the particle size less than 125 nm with PDI about 0.21, a good negative charge (−15.37 ± 0.91), excellent encapsulation efficiencies (94.89% ± 1.83%), and a sustained release for 48 h in vitro. Intracellular uptake of RGD-SSLs-SHK was higher than that of SSLs-SHK in the MDA-MB-231 cells, and the results of cytotoxicity confirmed the superior therapeutic efficacy of RGD-SSLs-SHK in vitro. Moreover, compared with SSLs-SHK, RGD-SSLs-SHK could greatly induce the apoptosis of MDA-MB-231 cells by increasing the ration of Bax/Bcl-2, and inhibit the MDA-MB-231 cells proliferation, migration and invasion by reducing the level of MMP-9 and NF-κB p65. The anticancer mechanism of SHK is further clarified. Therefore, all encouraging results implied that RGD-SSLs-SHK may provide a promising platform for improving targeted therapy breast cancer.

## Figures and Tables

**Table 1:** Characterization of liposomes (mean ± SD, *n* = 3).

Liposomes	Size (nm)	Zeta Potential (mV)	PDI	EE (%)
SSLs-SHK	121.36 ± 2.89	−16.32 ± 1.45	0.208 ± 0.021	92.51 ± 2.55
SSLs-Cou6	119.96 ± 3.41	−16.01 ± 1.68	0.213 ± 0.023	92.71 ± 2.91
RGD-SSLs-SHK	117.53 ± 3.05	−15.37 ± 0.91	0.180 ± 0.015	94.89 ± 1.83
RGD-SSLs-Cou6	120.67 ± 2.63	−15.86 ± 0.93	0.201 ± 0.014	93.84 ± 1.41

**Table 2:** The half-maximal inhibitory concentration (IC_50_) of different SHK formulations on tumor cells (mean ± SD, *n* = 3).

Liposomes	IC_50_ (µM) in MDA-MB-231 Cells	IC_50_ (µM) in MCF-7 Cells
SSLs-SHK	10.92 ± 1.03 ^∆^	3.34 ± 0.18 ^∆^
RGD-SSLs-SHK	7.16 ± 0.62 ^∆^ **	2.96 ± 0.12 ^∆^
free SHK	4.92 ± 0.29	1.90 ± 0.11

^∆^ Compared with free SHK, *p* < 0.05; ** compared with SSLs-SHK, *p* < 0.01.
